# The impact of the COVID-19 pandemic on harm reduction services in Catalonia: the experience of people who use drugs and harm reduction professionals

**DOI:** 10.1186/s12954-022-00699-1

**Published:** 2022-10-26

**Authors:** Mar Bosch-Arís, Laia Gasulla, Teresa de Gispert, Lidia Segura, Joan Colom

**Affiliations:** 1 Public Health Agency of Catalonia, Epidemiological Surveillance Service, Barcelona, Spain; 2 Public Health Agency of Catalonia - Programme On Addictions, HIV, STI and Viral Hepatitis, Barcelona, Spain

**Keywords:** Harm reduction, COVID-19, Lockdown, People who use drugs, Drug professionals

## Abstract

**Background:**

Harm reduction services and professionals have had to reorganise and adapt to COVID-19 prevention measures while still ensuring health and social services for people who use drugs (PUD).

**Objective:**

To assess the impact of the COVID-19 pandemic on PUD and on the professionals who provide harm reduction services.

**Methods:**

A qualitative, exploratory, multicentre design was used. Two focus groups were held with harm reduction professionals, and 40 individual semi-structured interviews were undertaken with PUD in various harm reduction services in Catalonia. Interviews and focus group discussions were transcribed and analysed using thematic content analysis.

**Results:**

Harm reduction services adapted to the pandemic situation by employing methods such as reducing opening hours and closing drop in areas, along with health protection measures such as access control, which in turn led to stress among both professionals and service users. Despite the changes implemented, PUD continued to have access to sterile drug use equipment and methadone treatment. In addition, those who were not in treatment were able to access it rapidly. Regarding their emotional state, the PUD reported that it was worse during the pandemic than before the lockdown, with women affected to a greater extent than men. The harm reduction professionals reported difficulties in managing service users’ compliance with the security measures at the beginning of the lockdown and having had to focus primarily on providing food and shelter for the PUD.

**Conclusions:**

It is important to keep PUD in mind and maintain a harm reduction perspective when implementing confinement measures in situations such as those experienced during the COVID pandemic. Guaranteeing that PUD have their basic needs such as food, hygiene and shelter covered is key.

## Background

On 31 December 2019, the first case of SARS-CoV2 (COVID-19) was reported in Wuhan, China, and the World Health Organization declared the COVID-19 pandemic on 11 March 2020. By then, the illness had spread to almost all countries and infected 20 million people [1].

The COVID-19 pandemic created an exceptional situation, which has present and future implications for different areas of society such as physical and mental health, the social environment and education.

To control the spread of the disease, Spain declared a first State of Alarm on 14 March 2020 (Royal Decree 463/2020) which established lockdown measures to prevent transmission and to avoid health system collapse. The State of Alarm lasted until 26 June 2020.

The measures adopted in response to COVID-19 had an impact on the day-to-day lives of people who use drugs (PUD), especially those who use harm reduction (HR) services, those in vulnerable social situations and those with chronic conditions and/or illnesses related to the immune system. Harm reduction services for PUD are a front-line public health interventions. They serve a population that due to stigma, discrimination and criminalisation, face barriers to accessing health and social services and are particularity vulnerable in public health crises [2]. The lockdown measures limited access to substances and also access to resources, including HR services [3, 4]. Furthermore, sociodemographic factors often seen among PUD, such as a low level of education, low economic status, unemployment, or not having stable housing, are related to difficulties in accessing treatment services [5, 6]. People who use drugs are also stigmatised and face discrimination, well-known barriers to accessing to treatment, health care and social services [7, 8]. This stigma is more pronounced in women who use drugs and further limits their access to services [9].

Harm reduction services specialise in the care of people who use high-risk drugs, such as injected or inhaled heroin and cocaine. These centres provide users with social and health care services from a public health perspective, with the aim of reducing the negative impact of drug use on physical and psychosocial health, facilitating access to treatment, and minimising the effects on the community of drug use in public spaces [10].

Fundamental to the effectiveness of these centres in reducing health risks for PUD (e.g. needle sharing, overdoses, infectious diseases such as HIV and Hep-C) is their accessibility and hours of coverage [11–13].

The main services offered are needle and syringe programmes, prevention and management of overdose, and health and social services (e.g. infectious disease screening, health education, prevention of sexually transmitted infections, hygiene and nutrition) as well as supervised drug use. In many HR centres, there is a space known as a drop in area where users can relax, get a snack and participate in activities like reading or board games.

Catalonia has led the implementation of harm reduction policies in Spain since the 1990s and currently (2022) has 18 HR centres, 8 mobile units, 13 street education services and 15 supervised drug use rooms and 1 hostel for PUD with a supevised drug use room [14]. Catalonia is a politically autonomous region in the north-east of Spain, and it has 7.7 million inhabitants. In 2020, HR services in Catalonia distributed 549,175 syringes and attended 5908 PUD [15].

During the pandemic in Catalonia, HR services and professionals had to reorganise and adapt to COVID prevention measures, while simultaneously ensuring health and social care for PUD. The situation created much uncertainty as to whether PUD, in a vulnerable situation, would have more difficulty than the general population in complying with the pandemic measures and accessing HR services.

## Objective

The primary objective of this study was to assess the impact of the COVID-19 pandemic on PUD and the professionals working with them in HR services.

## Methods

### Study design

A qualitative, multicentre, exploratory study using a hermeneutic phenomenological approach. To produce a theory based on interpretation, a dialogue was established between the participants and investigators; in that way, the interpretation of the data is not only descriptive, but is also grouped thematically and interpreted.

### Recruitment and sample

To ensure including as many points of view as possible, and to guarantee the heterogeneity of conceptual meanings in the sampling method, different criteria for PUD selection were taken into account: gender (male or female), country of origin (Spain and other countries), and housing situation (fixed place of residence, without home or with other type of housing). The PUD were identified by professionals working in HR services throughout Catalonia, including harm reduction centres, supervised consumption rooms (injected and inhaled), mobile units and outreach teams. It was considered important to obtain the points of view of PUD from different populations, to include different sociodemographic profiles (urban vs. rural), different consumption patterns and different levels of access to health and social services. In addition, effort was made to ensure that women were represented in numbers similar to that in which they attend HR services [9].

Inclusion criteria for PUD participation were: adult (> 18 years) drug users. Potential participants were excluded based on the following criteria: being highly intoxicated at the time of the interview or suffering serious psychiatric symptoms that impeded the interview, and people with difficulties understanding the language. Interviews were conducted in either Spanish or Catalan depending on the language preferred by the PUD and were translated into English.

A total of 40 PUD (15 women; 25 men) were recruited from 13 distinct HR services.

Harm reduction service professionals were recruited to the focus groups opportunistically. They were randomly selected from each HR service. Thirteen HR services participated in the study, and one professional was recruited from each centre (5 men; 8 women).

Prior to participation, both the PUD and the HR professionals were informed of the purpose of the study and informed consent was obtained from each participant. To ensure confidentiality, names were coded and identifying data was anonymized. The study protocol was approved by the ethics committee of Hospital Clinic, Barcelona (Reg. HCB/2020/0761). No incentives were offered for participation in the study, neither to the PUD nor the HR professionals.

### Data collection

Individual semi-structured interviews using open-ended questions were undertaken with the PUD to record their impressions and the changes experienced due to the COVID-19 pandemic. Interviews were undertaken by professionals working in HR services in distinct parts of the region in June 2020.

The interviews collected information on PUD’s views in relation to accessibility of HR centres, difficulties in following lockdown regulations, drug consumption, access to other health and social services or housing, relationships with the community, and their emotional state. A guide was developed to facilitate the interviews (Annex 1) and emotional state was evaluated using the validated WHO-5 Well Being Index [16, 17].

The impressions of and changes experienced by the professionals during the pandemic were collected via two focus group sessions. The focus groups were held by video call using Microsoft Teams due to the COVID-19 restrictions in place at the time. In the sessions, the impact on the HR professionals, with regard to available resources, coordination with other services, relationships with the local community, and service users’ adaptation to the lockdown related measures (including the impact on their emotional state) were evaluated. A guide was developed to facilitate moderating the focus groups (Annex 2).

### Data analysis

Both the interviews and the focus groups were recorded, with the prior consent of the participants. The recordings were transcribed to obtain the text for analysis. Preconceived ideas were identified and thematic content analysis was done using AtlasTi. During the analysis, the text was coded and grouped, obtaining emerging categories. These groupings were discussed among the researchers, thereby undertaking researcher triangulation.

## Results

The characteristics of the participating PUD are described in Table 1. Most participants were from Spain (76% of men; 67% of women), and they used a wide variety of drugs. (The table only includes the drugs or the drug combination that participants mentioned.) The primary method of drug use differed between men and women: for men the primary method was injection (52%), whereas for women the primary method was smoking (53%). Most of PUD interviewed lived in their own home (44% of men; 40% of women) and received income support (64% of men; 67% of women).

**Table 1:** Participant characteristics

	Men (*n* = 25)		Women (*n* = 15)	
	*n*	%	*n*	%
*Country of birth*				
Spain	19	76	10	67
Algeria	1	4	0	0
Russia	1	4	0	0
Mali	1	4	0	0
Lithuania	1	4	0	0
Brazil	1	4	2	13
Not stated	1	4	1	7
Portugal	0	0	1	7
Italy	0	0	1	7
*Main drug used*				
Heroin	9	36	5	33
Cocaine	8	32	4	27
Heroin + Cocaine	4	16	3	20
Alcohol	2	8	0	0
Benzodiazepines and cannabis	1	4	0	0
Cannabis	0	0	1	7
Crack	0	0	1	7
Not stated	1	4	1	7
*Primary method of drug use*				
Injection	13	52	4	27
Smoked	7	28	8	53
Oral	3	12	0	0
Sniffed	1	4	2	13
Not stated	1	4	1	7
*Living situation*				
Home	11	44	6	40
Street	6	24	2	13
Squatter	5	20	4	27
Hostel/refuge	2	8	3	20
Not stated	1	4	0	0
*Income status*				
No income	8	32	4	27
Pension/income support	16	64	10	67
Active (working)	0	0	1	7
Not stated	1	4	0	0

Following triangulation of the results of the interviews and focus groups, the categories were grouped into the following seven thematic areas.

1. Organisational changes in the HR centres

Both the PUD and the HR professionals perceived changes in the centres, such as closing of drop in areas, having to enter the supervised consumption rooms individually, and changes in the centres’ hours.

Um… no, well, the only thing is that, before we could enter in twos, now it’s only one at a time and this is a bit of a problem for us, because it’s hard for us to get money and sometimes we share. The fact that only one person at a time can enter…it creates more anxiety believe it or not (Female drug user).

The need to enter the HR centres individually created crowds at the entrance, which in turn created difficulties for the professionals in managing these situations. And also it brought an increase in no supervised consume, because PUD did not want to wait to enter to HR centres and started to consume in other locations.

Many times PUD share the substance... then what we have found now is that there are many, many people consuming on the street, many more. Many times they share or they come to the centre and there is someone else inside the consumption room and they don't come back... they take the needle and the syringe and leave. They do not want to be waiting outside, no... and they consume in front (Female HR professional).

Another change in the centres noted by the HR professionals was the stopping of regular activities and workshops.

All the workshops which we did were redefined and redirected due to COVID. From having to wear masks, provide information, make informative videos etc. Also because… the people who came … there wasn’t much awareness of what it was, there wasn’t much awareness… and so we focused a bit on working on this… (Female HR professional).

Stopping these activities worried the professionals because of the loss of contact with the PUD during the lockdown.

Us, what we did, what worried us most was being able to keep the group active, no? They are very intermittent… that we know, and the fact that the group wouldn’t have the continuity that we had had in the previous months worried us… What we did was…make a lockdown Whatsapp group, and we put challenges trying to follow the daily routine no? A trying to put challenges in Whatsapp (Female HR professional).

The HR centres had to make organisational and structural changes and kept contact with some PUD through digital tools. The PUD appreciated the professionals keeping contact and having received calls during the COVID-19 pandemic.

The treatment from the professionals… super supportive, calls, asking me how I was … If I had any… any problem or anything, I could send a WhatsApp even if it was 11 at night (Female drug user).

2. COVID safety and protection measures

The HR professionals described anxiety due to the changes in guidance regarding personal protective equipment (PPE) during the first months of the pandemic.

The hospital guidance … and the guidance for PPE in the hospital changed so many times… the level of uncertainty was terrible… because at the beginning everyone told us ‘only gloves, no mask, no nothing’… and we started the pandemic working with gloves but no masks. Of course, since then…um … now masks…now gown…now face shield…but it has been difficult… (Female HR centre professional)

The HR professionals also stated that not all of the PUD respected the safety and protection measures (mask and distance), even though in the interviews the PUD said that they knew what the measures were.

Of course, we have a van, we open it at the back, and we had to take a table and some safety barriers and make a space for ourselves. Although they entered, you turned and there would be someone there and you said ‘Don’t you see I’ve made a space here that you can’t enter?’ It was very difficult… (Male HR centre professional)

Well, I don’t know, I try to not get close to people, wear the mask, avoid… people who you can say are a bit unwell, who are coughing and whatever… keep 4 or 5 metres away in case they’re infected, who knows? … or it could be that they have a bad chest because they’re a smoker… but the coronavirus, I have no idea what side effects it has or anything … I washed my hands straight away when I entered, I wash them 4 or 5 times, I wash them with soap… (Male drug user)

At the beginning of the pandemic, the HR professionals had a sense of being unprotected due to the lack of diagnostic tests and anxiety in case one of the PUD tested positive. Although they had this feeling, they knew the process if there was a suspected positive case among the PUD. Among the PUDs interviewed, none had a positive PCR test.

Yes, we would have appreciated it, a little, to be able to do tests right? Because, honestly we didn’t understand if… well, if someone had had it or not… (Female HR centre professional)

Well, I agree completely with what was said… when we… if there had been a positive case, being in a primary health centre, it was easy to manage from the first moment… We talked about it and… well, in the end it always depends on the professional, the fear they have, and the stigma…it adds to our users… The care has been more or less good, but yes we had the system very… we had it from the beginning and very quickly. (Female HR centre professional)

3. Changes to consumption and access to drugs

Professionals perceived changes in consumption among the PUD during the lockdown; some increased their consumption while others reduced their consumption.

This, yes, um… normally, they use in pairs, 2 or 3 people come together, and we’ve noticed a change to individual use, right? (Male HR professional)

I think it depends on the circumstances. We have already seen, that since the new reality… excuse me, the new normal… people started to consume more. From that moment, they had already started. (Male HR professional)

Look, in part COVID was good, because I got used to using less, although I suffered a, really severe depression, really bad, it was terrible, but in a way it was good for me, that those around me weren’t selling, because I got used to using less and now I’m at a point where I hardly use at all. (Female drug user)

The professionals also noticed a change in methods of use; they stated that injecting drug use declined and inhaled drug use increased.

For example, now it’s not just the lockdown, yes there’s been considerably more smoking of drugs, more than injecting lately, right? In fact, we run out of foil, we don’t have enough, this is a sign that people are smoking more. Traditionally there was more cocaine injecting and people started to smoke more, and it could be that during the lockdown it was more noticeable (Male HR centre professional)

Even so, the HR centres continued to offer the needle and syringe programme, and in some sites, the number of syringes distributed increased. The professionals also observed an increase in benzodiazepine use, realising that they were easier for the PUD to access.

I think it’s been … not because they couldn’t find where to buy, but rather because of money… a hit of heroin and two benzos is easier, the effect is faster, than three hits of heroin… It’s also true that a prescription for benzos is much easier to get, in both the drug treatment centres [CAS] and Emergency... Because they go and … ‘no, it’s just that I’m really anxious’, ’I have, I don’t know what’, and bam…benzo. ‘And … drug user’ - benzo after benzo. And… it’s an impression… I don’t know numbers … But I think that if we looked at the benzos prescribed in the last three months, uhm… they’ve increased a lot. (Female HR professional)

PUD had problems accessing drugs, they were afraid to go out to the street to buy; people weren’t allowed to be on the street. Another factor that made access difficult, as much for PUD as for professionals, was the irregular hours at which drugs were sold.

Yes, partly yes, for me it’s that… something happened… I used less during this time because those near me stopped selling for three months because of fear of COVID, they stopped for three months … And because I didn’t dare go out much during these months I used less, but I had more cravings, that’s for sure. I used less because of this, because my usual seller stayed much further away and I didn’t dare go out much, truly, it was because of this. (Female drug user)

The PUD also described difficulties in getting money to buy drugs, for those with no income or pension it was really hard.

Also, it’s really hard to get money when there’s no one on the street, when all supermarket is closed, everything… you don’t have many options to make money in the street, and this is hard, for example, you could find a kilo of gold on the ground but you don’t have anywhere to sell it. So you have nothing. (Male drug user)

Of course, because I beg in supermarkets, now there are not people in the street, you can hardly get any money; people don’t have coins to give you because they are only using cards. As for prostitution, I do not trust it either, and the people… It’s that people don’t trust… everything at once. (Female drug user)

Regarding the quality of drugs, both the PUD and the professionals agreed that purity and quality declined during the lockdown.

Most of the PUD interviewed before the lockdown were already in treatment with opioid agonists, and according to the professionals participating in the study not many new patients entered treatment during this time.

Regarding treatment initiated during the lockdown, both the PUD and the HR professionals highlighted the rapid inclusion of interested people into the programme. Both groups also described changes in methadone dispensing; it became more flexible and treatment was provided for longer periods to avoid movement and social interactions.

Because I’m in the Badalona centre, in the Delta, at that time… Well yes, they put you on methadone, but there they made you go one at a time. I thought that was really good because there’s less risk of getting COVID, so they made you go one at a time and wash your hands with that liquid, and you went there to take the medication. They gave me the medicine twice a week and so I went there took the medication and went back two or three days later (Male drug user)

4. Access to other resources

The PUD and the HR professionals agreed that it was impossible to admit people to therapeutic communities (long-term residential treatment for substance use disorders) and emphasised the need to ensure access to therapeutic communities in the case of another situation like the lockdown.

Um … regarding the users I felt really powerless because we had users who really needed to be admitted… well … some were in the process of being able to enter detox, others were waiting admission to therapeutic communities… There are people who have returned to using in huge amounts… we told them they needed to stop… and it was really hard to manage people in this situation (Female HR centre professional)

For the HR professionals, one of the biggest concerns during this time was the difficulty in providing shelter for the PUD. Those who had managed to access hostel services complained about the entry and exit restrictions and the rules that the general hostels imposed; they agreed with the professionals that these should be modified in the future.

One user who … they complained a lot about the hostel, the municipal hostel, that they didn’t consider the characteristics of PUD and they were continuously expelled (Male HR centre professional)

Overall, we saw that the first hostels that opened were very restrictive, no? With rules… That were unnecessary, no? If you came from the street and… you only have a backpack, it is not normal that they only let you access your things once a day. Because those are the only things you have. These small details could make the difference between whether someone can stick with it or not. (Female HR centre professional)

They didn’t let me in until I was on the street and had got sick, low defences and nothing… bad, terrible. Because now they tell you that there’s priority. Twice I went to talk with the assistant and they told me it was completely full, no vacancies, and that I had to show proof that I’d been here in Lleida for two months, but I am from Lleida. So, nothing. Nothing, really bad, really bad social services. (Female drug user)

Most of the hostels and resources did not take into account the specific needs of PUD during the lockdown, and the rules and strict norms were a problem for PUD during their stay. But in Barcelona, one hostel addressed to PUD was opened, and some of the problems that PUD had in other hostels were solved with this one resource. Both groups agreed that that the implementation of a hostel aimed at drug users in Barcelona was very satisfactory:

Yes, I went to Barcelona… I had a problem here in Terrassa, to do with abuse of tranquilisers and stuff. I spoke badly, um… to the face of people who you shouldn’t talk bad to and I had to move to Barcelona and I was housed immediately in Barcelona, in the COVID centre… and the experience was really good, and the treatment I received was really positive… So, I really appreciate what the authorities did for homeless people, because in the end a homeless person, um… they’re rarely going to get the virus and pass it to too many people because they don’t have many relationships. I think it was really good that they spent this money for homeless people. (Male drug user)

Some PUD were able to access food and sanitary services offered by the HR centres. The professionals stated that guaranteeing food for the PUD was one of the main concerns they had during the lockdown. Additional places were made available in the soup kitchens for PUD, and some HR centres incorporated a food service.

Um… after… What worried us most of all was the issue of food, food for guys who collect scrap metal, uh… that don’t have food. That is, they say ‘No, it’s that I don’t want to use, it’s that I want to eat’. So, obviously… we had to buy bottles ….gas bottles… and in the end we managed to get boxes of food from the Red Cross here in Hospitalet, because it was, uh… it was impossible, impossible to find any other way. So, well… on that hand, great. (Male HR centre professional)

5. Social and community relations

On a few occasions, during the lockdown, the police fined PUD for breaking the lockdown rule and being out on the street. The HR services professionals noted that it seemed that the PUD had more problems with the police during this time.

Because when were in a total state of alarm, the police couldn’t see you in the street, and sometimes, when you’d snuck out to score they stopped you and asked where you’d come from and where you were going, and they found me a few times. (Female drug user)

The HR professionals highlighted the role of the local community during the lockdown. In some parts of the territory, they felt great support from the local community. However, in other parts of the territory, it was the opposite; for example, in one area opposition from the neighbours stopped the opening of a hostel for drug users.

And… for us, it was nice because it created a really strong connection between the women and the community. There was a sense of brotherhood among the support groups in Raval (Female HR centre professional)

But with the neighbours completely against it, that is, we had constant complaints from the neighbours. Neighbours shouting at the door, well… The bad feeling of the local neighbours continued, from the protests we had at the beginning of the year until now, they continue… to the point that in full lockdown the neighbours protested against the opening of a hostel and the mayor decided that it wouldn’t open due to the pressure from the neighbours (Female HR centre professional)

6. Emotional state

The emotional state of both the HR professionals and the PUD was not consistent. We found some PUD had taken a positive attitude towards the lockdown and had used it to create a new routine. On the other hand, some said they had been sad and depressed and had been afraid of infecting those close to them.

Awful, really really bad, I got an extreme depression, that I’d never had, but with all the people who were dying, worse, because my mother died not long before, because I spent all day watching TV, all day talking about the same thing, um…so. Emotionally I was worse than ever, I think. Worse than ever. That’s the truth (Female drug user)

Table 2 shows the results of the WHO-5 Well-Being Index [17]. The average result for men was slightly higher than for women. However, the percentage of PUD with clinical depression was higher in men than in women. There was a considerable variation in responses from one person to another, and in mood depending on the person and how they approached the lockdown.

**Table 2:** Results of the WHO-5 Well-Being Index for PUD

	Global	Men	Women
Average score	44.2	49	45.5
% PUD scored < 50 Cut-off for clinical depression	53%	63%	46%

Among the HR professionals, the majority feared either themselves or their family catching the illness. They were also affected emotionally by being away from their families and by long periods of time without seeing them. On the positive side, they felt that they worked as a team with their colleagues to overcome the challenges they faced during these months.

On a personal level, those first days … a lot of fear, it complete paranoia, or whatever, arriving home like ‘don’t touch me’, no? Quick shower, disinfection, take my temperature, buy another thermometer because I didn’t trust the result of the first, a bit paranoid … I was a bit paranoid… (Female HR centre professional)

And… at the personal level, well, I’m also from Tortosa, and obviously, 5 months alone at home, without family, videocalls with my mother, which I’d never done and things like that, well it was really hard. And now, after 5 months I’ve been able to go down there for 5 days. (Male HR centre professional)

7. Suggestions for improvement

During the focus groups sessions, the HR professionals made suggestions for improvement in the case of a possible future lockdown. The thing that they agreed on most was covering the basic needs of the PUD, with a special emphasis on housing and food. They also stated that all the services that were put in place during the lockdown should be maintained although there was no longer a lockdown (soup kitchens, hostels, etc.).

I think that, well, improve or keep in mind what worked, the resources used or what was implemented and worked, and to be able to continue with these resources whether it’s a lockdown or not (Female HR centre professional)

They also agreed on requesting that the hostels be equipped with a space for supervised drug use with professionals to attend the drug users with a harm reduction approach.

They believed it would be appropriate to improve communication between the different HR services in Catalonia by creating a channel for sharing information about available resources (hours, accessibility) to enable rapid referral for the PUD.

And I think that it would have also been very useful for us to have some kind of channel to be able to update which of the services were maintained and which were closed … because it was, ‘is the drug treatment centre [CAS] open?’, ‘Which centre is open, and which not?’ And you had to go looking at them one by one… (Female HR centre professional).

## Discussion

The main changes in the HR services resulted from the reduced opening hours and closure of the drop in areas, and from the protection measures (such as having to enter one by one) which created stress for the PUD. These restrictions had an impact on the core features of the services—coverage and accessibility.

The PUD had more difficulty accessing drugs during the lockdown. Nonetheless, they continued to have access to hygienic drug use equipment and to opioid agonist maintenance treatment.

Not all the PUD had access to residential services or access to housing, food and hygiene, issues that were one of the main concerns of the HR professionals during the pandemic [18].

Compared to before the pandemic, the PUD’s emotional state was in general worse, more notably among the women.

HR professionals cited coordination problems at the beginning of the lockdown, and their primary concern during this time was ensuring housing and food for the PUD.

The results of this study are consistent with another study, which found that 84.6% of HR centres in Spain kept their services open [19] with some changes in hours, but that they continued offering needle and syringe programmes, access to supervised drug use rooms and other programmes.

The measures adopted by HR services as entering one-by-one to the consumption area has brought an increase in consume in locations outside of HR centres, no supervised consume and it could have an increase in overdoses. More research is needed to explore this possible association.

The increase in inhaled drug use referred to by the HR centre professionals was a trend that could not be attributed to the pandemic given that it had already been observed before the pandemic began. An increasing trend towards inhaled use and a reduction in injecting has been observed in Catalan PUD since 2017, and between 2018 and 2019 use by inhalation doubled [20].

The increase in the distribution of sterile drug use equipment by the needle and syringe programme reported by some of the HR professionals is not a trend that has been otherwise observed. In 2020, across all Catalan health services offering a needle and syringe programme, the distribution of syringes decreased by 22% compared to 2019, and in HR services it decreased by 27% [21].

Although measures to restrict movement were implemented, the PUD reported that they were able to access drugs without much difficulty during the lockdown. They reported that prices were maintained but quality declined. Nonetheless, according to the research, there was no interruption to supply, nor did the purity change significantly [22].

The PUD interviewed felt that the process for starting maintenance therapy with opiate agonists was more agile, highlighting above all, the speed of initiation.

As seen in the earlier literature, where PUD live has an impact on their access to HR services and treatment [23]. We found differences in access, both to substances and to resources, between PUD who lived in urban areas (namely Barcelona) and those who lived in rural areas further from Barcelona. One clear example is the provision of hostels for drug users, which gave those PUD in Barcelona and surrounding areas access to shelter and the ability to use drugs in a safe space. In other parts of the territory PUD had many problems due to using in the hostels, which had not considered the characteristics of people with active drug use, and this led to continual expulsions, restricting going out, and very strict rules which most of the PUD could not adhere too.

For the HR professionals, ensuring housing and food was a primary concern. The HR services considered the limited access to social services for PUD to be the most problematic [19], followed by the difficulties the PUD had with the police in the street (including fines and persecutions in some cases). The COVID-19 pandemic and the mitigation measures taken contributed to increasing and making more evident the socio-economic inequality which exists among PUD [24].

At the beginning of the pandemic, the HR professionals experienced a chaotic situation due to changing criteria and a sensation of being unprotected (e.g. lack of equipment, lack of clear guidance, etc.). They had to reorganise the HR services to continue to attend to PUD while adapting them to comply with the current measures. During this period, cohesion in the professional teams which enabled them to move forward and continue to provide essential services for PUD stood out.

### Limitations and strengths

The main limitations of this study are related to the difficulty of extrapolating the results to other populations of PUD. The sample used can only capture the experiences and perspectives of the people who chose to participate, and these could be more inclined to report on the impact of the pandemic, and this is not a representative sample.

One of the strengths of the study is that it analysed the direct and indirect impacts of the pandemic on a very concrete population, PUD. It also allowed for an examination of how Catalan HR centres were impacted.

In addition, this study was undertaken using rigorous qualitative research methods. The context of the participants was taken into account; inconsistencies, contradictions and exceptions which confirm the results were sought; different actors were included; results of subgroups were compared with the available literature; and finally a triangulation of methods (data obtained by different means) and analysts was undertaken.

## Conclusions

It is important to consider PUD and a harm reduction perspective when applying lockdown measures or restricting movement in crisis situations such as the COVID-19 pandemic, as these measures led to limiting accessibility to and coverage of harm reduction programme. During the lockdown, the need to prioritise covering the basic needs of PUD such as food, hygiene and housing was made clear. The experience of the residential services where drug use was allowed was highly regarded by both the HR professionals and the PUD and affirms the need for the permanent establishment and enlargement of these types of services.

**Fig. 1:**
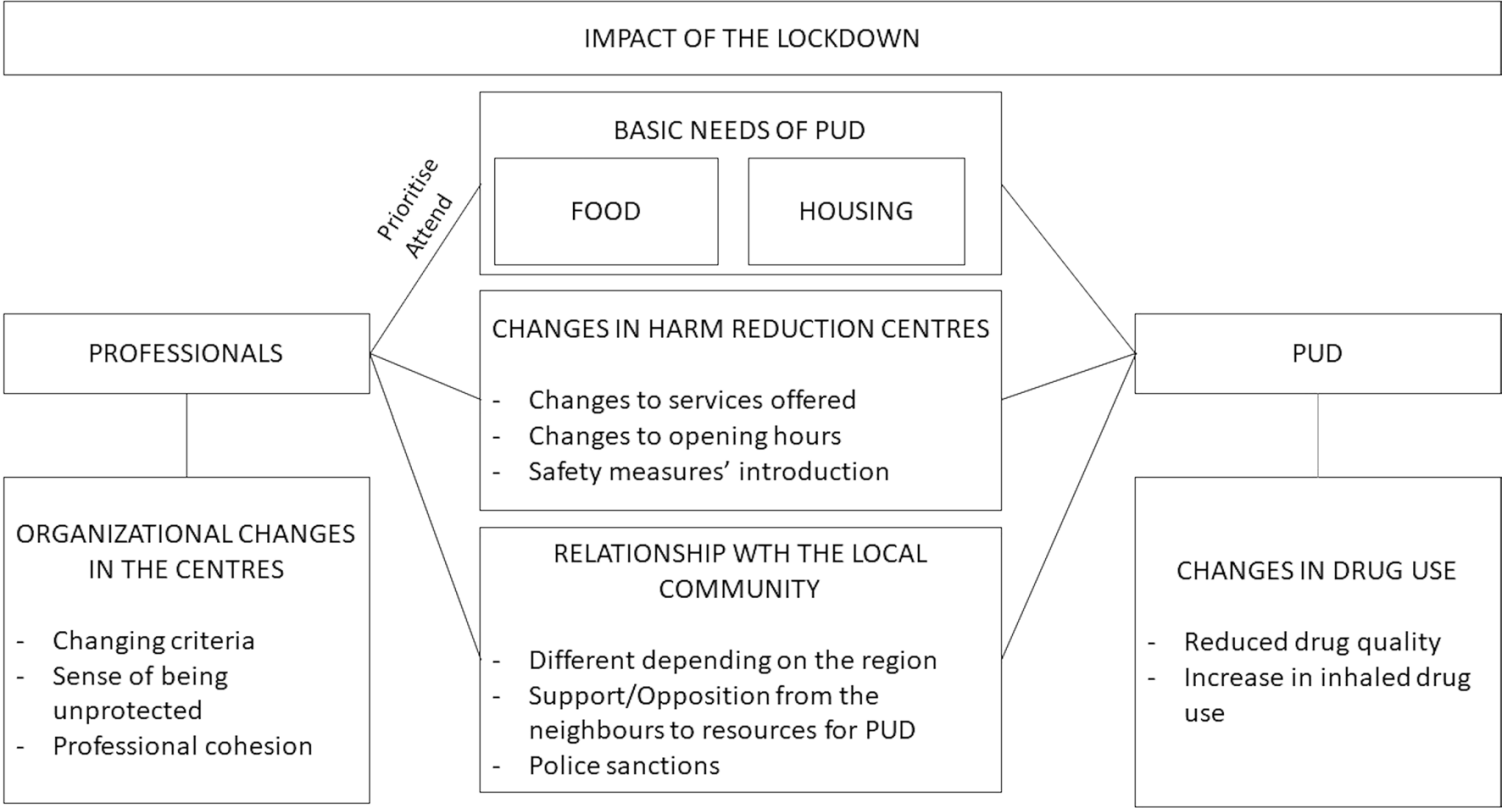
Impact of the lockdown

The lockdown period highlighted even more the fact that some basic needs (housing and food) of the PUDs are not covered. It is necessary to develop a comprehensive approach that enhances attention focused on the person and adopt intervention measures that address the different areas (social, health, psychological, etc.) with the objective of breaking these inequalities and promoting the general well-being of the person. For this, it is necessary to work in an interdisciplinary and coordinated way between the different resources involved both in the field of drugs and others in the health and social system (Fig. 1).

On the whole, HR services are essential to guarantee access to treatment, health care and social services and are able to engage a population that other services fail to reach. Governments must recognise and protect services that address the needs of people with limited access to health and social services.

## Data Availability

Not applicable.

## Annex 1: Guide for individual interviews

### Individual semi-structured interview

The objective of this interview is to identify and evaluate to what extent the current crisis, provoked by the COVID-19 pandemic, affected different factors related to the use of drugs, access to harm reduction services and other health and social services, as well as the emotional state of the interviewees. It also aims to obtain information about the person’s awareness of the COVID-19 security measures and whether these have been incorporated into their drug use.

To achieve this, it is proposed to undertake this semi-structured interview with drug users who visit different harm reduction services in Catalunya. The interview consists of 2 parts:

(I) Introductory questions
(II) 17 open themed questions, structured around 4 domains:
  - COVID-19
  - Access to harm reduction and other services
  - Drug use
  - Emotional state

The interview is recorded (audio)

*****IMPORTANT*****

- Ask permission to record the interview and reassure that information will be treated in confidence and anonymity will be maintained
- Interview structure:

**Table:**

1. Welcome
2. Introduction
Thank for participation
Explain the reasons for and objectives of the study
Explain that audio will be recorded
Request explicit permission to record the interview and reassure that information will be treated in confidence and anonymity will be maintained
Describe the characteristics and the reasons for the interview
3. The semi-structured interview
Introductory questions
Thematic questions
4. Close the interview
Summary of issues discussed
Final considerations
Explain how and for what the information will be used
Thank the interviewee for their participation

Service or entity __________________________

Gender: ______________

Country of birth: _______________

Main drug/s used: ________________________

Main route of administration: ____________________

Living situation/housing: ___________________

Main source of income: ______________________

1. Have you had symptoms related to COVID-19? If yes, was it confirmed with a positive test?
2. Would you know what to do if you had symptoms? If yes, briefly describe what you would do.
3. If you have done a COVID-19 test, what was the result?
4. Do you know the general recommendations for preventing COVID-19? If yes, please describe those that you know
5. Since the COVID-19 outbreak, have you noticed any changes in the harm reduction services? If yes, describe these related to:
  - Centre hours
  - Services offered (needle and syringe programme [PIX], drop in area, consumption rooms, hygiene, food)
  - Access to assigned professional
  - Treatment by professionals
  - Access to naloxone
  - Other
6. During the COVID-19 crisis has it been more difficult to get injecting equipment or other drug use equipment than before?
7. For injecting drug users. Have you had to go to a different needle and syringe programme (PIX) service than your usual? (If yes, specify)
8. For people who inject heroin. Have you considered starting - opioid substitution treatment? If yes, do you think you would you be able to access it quickly and easily?
9. If you needed them, were you able to access social and health services during the COVID-19 crisis? (housing services, food, legal services, hygiene…)
10. How did the lockdown affect your ability to make money?
11. Where did you use drugs during the complete lockdown? Did you do it with others?
12. Did you have problems accessing drugs?
13. Was there any change in access to drugs compared to before COVID-19, in terms of distribution, price, purity or the amount you could get?
14. Because of COVID-19, have you taken any safety measures related to your drug use? Could you describe some of them?
15. Have you had problems with the police, neighbours, friends/associates etc.? If yes, briefly describe them
16. How did you feel during the lockdown? (*collect information about the person’s mood in relation to the period prior, during, and after the lockdown*)
17. In the following table, for each of the 5 statements, please indicate which best describes how you’ve felt during the last 2 weeks.

Note that larger numbers indicate better well-being. For example, If you felt “cheerful and in good spirits” more than half the time, during the last 2 weeks put a cross in the top left corner of the third box.

Once completed, read the responses and scores out loud. For example, Question 1. I felt cheerful and in good spirits. Score 3.

**Table:**

During the last 2 weeks:	All of the time	Most of the time	More than half the time	Less than half the time	Some of the time	At no time
I have felt cheerful and in good spirits	5	4	3	2	1	0
I have felt calm and relaxed	5	4	3	2	1	0
I have felt active and vigorous	5	4	3	2	1	0
I woke up feeling fresh and rested	5	4	3	2	1	0
May daily life has been filled with things that interest me	5	4	3	2	1	0

## Annex 2: Focus group discussion guide

INTRODUCTION

1. Welcome the participants
2. Explain the reasons behind the session
3. Explain why they were chosen for the study
4. Explain the dynamics of the session
  - Tell them that the session will be recorded
  - Request explicit permission to record the focus group discussion guaranteeing confidentiality and anonymity of the information. When transcribing the session, personal information will be coded to avoid identification.
  - If they all agree, start to record the session
  - Make it clear that there are no right answers only different opinions, both negative and positive responses are important
  - Emphasise that the aim of the session is to collect information about how they were affected by the pandemic and what are their perceptions of the pandemic as professionals in harm reduction centres. It is not a session in which they should give their answers from the perspective of the Program on Substance Abuse, Department of Health
  - It is a space for reflection based on interaction between professionals.
  - To make a comment “raise your hand” or if not possible, ask in the chat.
5. Explain that the session will be transcribed, and the transcription shared with the participants so that they can make revisions, clarifications etc.
6. Ask if they have any questions before starting.
7. Prior to the focus group, all of the participants should have been informed in writing, of the objectives of the focus group, and have signed the form indicating informed consent.

SESSION STRUCTURE

**Question 1.** Many of you may know each other but not all of you. What do you think if we first do a round of presentations, saying our names, where we work and what work we do?

**Question 2.** One of the COVID-19 measures put in place by the government was the lockdown. This situation also led to a series of further safety measures. With this is mind, focusing on the lockdown period (March and April):

Could you broadly describe how you saw your centre/mobile unit affected during this time (hours, professionals, available services…)?

**Question 3.** In your experience, what difficulties were there in managing the centre during the lockdown (daily activities, managing drug users etc.)?

**Question 4.** Were there suspected or diagnosed cases of COVID-19 among the professionals and/or drug users? How were the cases managed? Did you find yourself in situations you didn’t know how to manage?

**Question 5.** According to your experience and opinion, how did the drug users react/respond to the changes produced by the lockdown (e.g. access to the centre, access to drugs, drug use patterns, following the established safety measures…)

**Question 6.** How would you describe the relationship you had with the drug users during the lockdown?

**Question 7.** Did you observe changes in your emotional state and your relations with other people (professionals, personal relationships)?

**Question 8.** How was the coordination with other services: social services, drug treatment centres, government agencies (Program on Substance Abuse, Dept. of Health, Department of Social Rights and Citizenship…)

**Question 9.** And with the community actors (police, neighbourhood…)?

**Question 10.** We would like to know your opinion on the specific recommendations from the Program on Substance Abuse, Dept. of Health

**Question 11.** What aspects do you think were overlooked during this time and what should have been prioritised?

**Question 12.** Would you like to add anything else?

END

**Question 13.** Does your impression of the session align with the summary we just made? Is there any information or relevant aspect missing? Or would you like to clarify any detail?

**Question 14.** To finish up, we’d like to ask you for a word/phrase which defines your main feeling during the lockdown; and another word/phrase for what is needs to be improved should we find ourselves in a similar situation in the future.

THANKYOUS AND CLOSE OF THE SESSION
